# Idiopathic catatonic syndrome in a young male with no prior psychiatric history: a case report

**DOI:** 10.1186/s13256-023-03903-3

**Published:** 2023-05-03

**Authors:** Jennifer L. Zick, Lora Wichser

**Affiliations:** 1 Psychiatry Residency Program, University of Minnesota, Minneapolis, MN 55455 USA; 2 Department of Psychiatry and Behavioral Sciences, University of Minnesota, Minneapolis, MN 55455 USA

**Keywords:** Catatonia, Case report, Differential diagnosis, Catatonia course, Electroconvulsive therapy

## Abstract

**Background:**

Catatonia is a syndrome characterized by severe psychomotor disturbances such as hypomotility, bradykinesia, and unusual movements. The condition has been described in the context of a wide variety of primary disease processes, including psychotic and mood disorders and numerous general medical conditions. In the medical community, catatonia is misunderstood, under-recognized, and under-treated. There continues to be debate about whether catatonia is an independent syndrome or a secondary expression of other conditions. This is a unique case presentation, as there are few reports describing cases of isolated catatonic syndrome in the absence of any other psychiatric or medical condition.

**Case Presentation:**

We present the case of a 20-year-old previously healthy Caucasian male whose initial presentation to psychiatric care was in the form of an acute catatonic syndrome dominated by mutism, blank staring, and poverty of movement. As the nature of the patient’s symptoms precluded the collection of a complete psychiatric  and medical history, we employed a broad differential diagnosis including catatonia due to another medical condition, catatonia as a specifier for a number of mental disorders, and catatonia not otherwise specified.

**Conclusions:**

The presentation of an acute onset of psychomotor symptoms in the absence of a history of mental illness warrants extensive workup to rule out medical causes to ensure effective treatment of any underlying illness. Benzodiazepines are the first-line treatment for catatonic symptoms, and electroconvulsive therapy can be used to resolve symptoms in patients who do not respond to medical intervention.

## Background

Catatonia is a unique neuropsychiatric syndrome consisting of altered consciousness, poverty of speech and thought, and striking psychomotor abnormalities. Catatonia was originally described by Karl Kahlbaum in 1874, who wrote of a cyclic, alternating disease process consisting of mood abnormalities, stupor, confusion, and motor symptoms such as catalepsy, mutism, muscle rigidity, and negativism [1]. Later descriptions by Kraepelin and Bleuler led to the categorization of catatonia as a subtype of schizophrenia, and the syndrome remained in this branch of psychiatric nosology throughout the 1900s and the use of the Diagnostic and Statistical Manual of Mental Disorders, fourth edition (DSM-IV) [2].

In the 1970s and 1980s, descriptions of catatonia recognized the presence of catatonic symptoms in a number of other neurological and medical disorders [3], prompting the inclusion of “catatonia due to a general medical condition” in the DSM-IV. Simultaneously, experts began to recognize that the majority of cases of catatonia occurred in the context of major mood disorders [4, 5]. In the DSM-5, catatonia is no longer listed as a subtype of schizophrenia, and serves as a specifier for a number of psychotic and mood disorders (Table 1). Catatonic disorder not otherwise specified (NOS) was also added to the DSM-5 to document the presence of catatonia without sufficient criteria to diagnose the disorders in which it can be utilized as a specifier. In particular, this was intended to account for presentations in which symptoms characteristic of catatonia cause clinically significant impairment but either the nature of the underlying mental condition is unclear, full criteria for catatonia are not met, or there is insufficient information to make a more specific diagnosis [6, 7].

**Table 1:** Catatonia diagnoses in the Diagnostic and Statistical Manual of Mental Disorders, fifth edition

The diagnosis of catatonia in DSM-5 (APA, 2013):
1. Specifier “with catatonia” when criteria are met for catatonia during the course of a psychotic, bipolar, depressive, neurodevelopmental, or other mental disorder
a. Schizophrenia
b. Schizoaffective disorder
c. Schizophreniform disorder
d. Brief psychotic disorder
e. Substance-induced psychotic disorder
f. Major depressive disorder
g. Bipolar I disorder
h. Bipolar II disorder
i. Autism spectrum disorder
2. Catatonic disorder due to a general medical condition
3. Catatonic disorder not otherwise specified (NOS)

In a longitudinal observation study of 180 episodes of catatonia in 148 individuals over a clinical follow-up period of 20 years, the distribution of concurrent or underlying diagnoses were as follows: affective disorder, 46%; schizophrenia, 20%; schizoaffective disorder, 6%; a range of medical/neurological illnesses, 16%; benzodiazepine withdrawal, 4%; and other psychiatric disorders, 8% [8]. Other studies have found that catatonic states induced by substances and general medical conditions account for 20–40% of cases of catatonia [9]. While cases of idiopathic recurrent catatonia have been described (for example, [10]), there is no form of primary catatonia currently recognized in the DSM-5 [11]. The present case is unique due in large part to the isolated catatonic signs and symptoms in the absence of any documented psychiatric history; to the authors’ knowledge, there are no such cases reported elsewhere in the literature.

It has been noted that catatonia often goes unrecognized by clinicians [12], particularly in cases that involve agitation, a combination of hyperactive and hypoactive behavioral patterns, or when catatonic symptoms are overshadowed by comorbid conditions [13, 14]. Recent estimates in multiple geographic regions suggest that approximately 9–15% of patients in psychiatric inpatient units display clinically significant features of catatonia [8, 15, 16].

In some cases the presence of catatonia is unmistakable and occurs in the near absence of significant features of any other psychiatric condition. In this situation, the patient’s care team is tasked with identifying the most likely etiology of the condition and pursuing the most appropriate treatment, often with the added challenge of collecting information from a minimally- or non-communicative patient. In these cases, the differential diagnosis must be broad to account for the many unanswered questions that can only be addressed with a more complete history. If a primary underlying etiology (general medical or psychiatric) is identified that is thought to have precipitated the catatonic state, treatment of this condition should be initiated. However, because the first-line treatments for catatonia are safe and effective, empirical treatment should be pursued in a timely manner.

## Case presentation

A 20-year-old Caucasian male college student with no known psychiatric history was brought to the emergency department by his roommates after isolating in his bedroom for 2 days with reduced motor activity and reduced verbal responsiveness. On admission, the patient was noted to have tachycardia and dilated pupils, and appeared to be distressed and preoccupied with internal stimuli. He also displayed severe psychomotor retardation, impoverished speech with an absence of spontaneous utterances, intermittent eye contact with periods of vacant staring, blunted affect, inability or refusal to follow commands, and apparent overall poverty of thought. Thought content could not be assessed. He lacked insight into his condition, and judgment and biorhythms were grossly impaired. Physical examination was negative for fever, neck stiffness, seizures, trauma, and focal neurologic deficits. Physical and psychiatric symptoms could not be assessed due to poverty of speech, though the patient’s roommates were unaware of any recent symptoms reported by the patient.

The patient’s medical history, obtained by the patient’s mother, included uncomplicated mild asthma without history of hospitalization, as well as anaphylactic allergy to a specific food. The patient’s mother was unaware of any history of symptoms that would be concerning for any psychiatric diagnoses, including mood or psychotic disorders. Family history was incomplete as the patient was adopted; however, there was an unconfirmed history of bipolar disorder in the patient’s biological father. Urine toxicology screen at presentation was negative for all tested substances. The patient's roommates reported occasional cannabis use by the patient, but not in the preceding several weeks. There was no known evidence of recent systemic illness or infection. The patient’s mother reported that the patient had visited the Rocky Mountains 2 months prior to presentation and had noticed a mild erythematous rash several days after returning. No further details of the rash’s appearance could be confirmed.

Lorazepam was initiated at 1 mg three times a day (TID) on hospital day 0, and risperidone was initiated the next day and maintained at 0.5 mg two times a day (BID) for another 12 days. The patient exhibited a partial response to lorazepam and was able to provide intermittent answers to questions about his mental state, revealing paranoia, sensory disturbances, a severely depressed mood, and passive suicidal ideation.

After 13 days, the patient was transferred to our facility. Clinical assessment confirmed that DSM-5 criteria for catatonia were met. Formal assessment with the Bush-Francis catatonia rating scale [17] revealed a severity score of 24 (out of a maximum total score of 69), based on the following characteristics: immobility, mutism, staring, posturing, stereotypy, mannerisms, negativism, waxy flexibility, withdrawal, impulsivity, automatic obedience, combativeness, and ambitendency (meeting DSM-5 criteria for a diagnosis of catatonia; see Table 2). He continued to appear catatonic, with severe psychomotor retardation, blank staring, and minimal responsiveness in the awake state. Risperidone was discontinued and replaced with quetiapine (300 mg/day) to avoid the possibility of worsening catatonic symptoms. We continued treatment with lorazepam 1 mg TID, which continued to have a minimal effect.

**Table 2:** Definition of catatonia in Diagnostic and Statistical Manual of Mental Disorders, fifth edition

Definition of catatonia in DSM-V (APA, 2013):
Catatonia is diagnosed when the clinical picture is dominated by three or more of the following:
1. Stupor: No psychomotor activity; not actively relating to environment
2. Catalepsy: Passive induction of a posture held against gravity
3. Waxy flexibility: Slight and even resistance to positioning by examiner
4. Mutism: No, or very little, verbal response
(not applicable if there is an established aphasia)
5. Negativism: Opposing or not responding to instructions or external stimuli
6. Posturing: Spontaneous and active maintenance of a posture against gravity
7. Mannerisms: Odd caricature of normal actions
8. Stereotypy: Repetitive, abnormally frequent, non-goal directed movements
9. Agitation (Not influenced by external stimuli)
10. Grimacing
11. Echolalia: Mimicking another’s speech
12. Echopraxia: Mimicking another’s movements

If 3 or more of the 12 psychomotor features are present, the specifier “with catatonia” can be added to the mental disorders listed in Table 1. These criteria can also be used to identify catatonia due to a general medical condition and catatonia NOS

The severity of the patient’s symptoms fluctuated during his hospital stay with variations in environmental factors and the time before and after administration of medications. Occasional episodes of relative lucidity revealed little about the patient’s internal state apart from severe confusion, disorientation, and anxiety. His affect was predominantly flat with some features of anxiety. The patient endorsed feelings of depersonalization and/or derealization, and several times asked his providers if he was dead. He also displayed prominent ambitendency, often appearing to experience debilitating indecision about trivial matters such as whether to stand or sit during a clinical interview. In retrospect, the patient later described his subjective experience as one of extreme indecision, apparently leading to an inability to settle on any particular train of thought or chain of actions. In contrast to the slowness of his physical presentation, in his words, “my mind was running so fast I couldn’t do anything.” He was unable to retrospectively identify the content of these racing thoughts.

### Investigations

Diagnostic workup performed during the patient’s initial hospitalization included urine toxicology screen, serum tests for alcohol and salicylate levels, metabolic panels, thyroid stimulating hormone (TSH), vitamin B_12_, methylmalonic acid, folate, ammonia, creatine phosphokinase, hepatic panel, and coagulation studies, all of which were within normal limits. The non-contrast head computerized tomography (CT) scan and non-contrast magnetic resonance imaging (MRI) scan of the brain were unremarkable, as were a routine electrocardiogram (EKG) and 20-minute video electroencephalogram (EEG; conducted during awake and stage 2 sleep stages with no evidence of underlying seizure disorder or focal neurologic dysfunction). The possibility of Wilson disease was assessed by serum ceruloplasmin and urine copper levels, both of which were within normal limits. Infectious and autoimmune etiologies were investigated with complete blood count, C-reactive protein (CRP), erythrocyte sedimentation rate, antinuclear antibodies (ANA), blood culture, and serum analysis for antibodies directed against *Treponema pallidum*, hepatitis C, and tick borne diseases (*Erlichia chaffeensis*, *Anaplasma*, *Babesia microti*, and *Borrelia burgdorferi*) in addition to a Lyme antibody immunoblot test.

The results of these tests were largely unremarkable, with the exception of a positive result early in the disease course for immunoglobulin M (IgM) antibodies directed against *Borrelia burgdorferi*, the pathogen most commonly associated with Lyme disease. The patient was evaluated by the infectious disease team, and a lumbar puncture was performed under anesthesia on hospital day 8. Cerebrospinal fluid (CSF) analyses revealed colorless and clear fluid at normal opening pressure with normal glucose and protein levels. Rapid polymerase chain reaction (PCR) analysis of the CSF was negative for Lyme disease pathogens (*B. burgdorferi*, *B. mayonii*, *B. garinii*, and *B. afzelii*), herpes simplex virus (HSV) 1, HSV2, enterovirus, varicella zoster virus, arbovirus, and several other viruses associated with encephalitis. CSF antibody panel was negative for antibodies directed against N-methyl-D-aspartate (NMDA), α-amino-3-hydroxy-5-methyl-4-isoxazolepropionic acid (AMPA), and γ-aminobutyric acid type B (GABA-B) receptors; the voltage gated potassium channel-complex (VGKC-complex), Glutamic acid decarboxylase (GAD65), and other proteins associated with autoimmune encephalitis.

### Differential diagnosis

At the time of presentation, the patient met criteria for catatonia NOS, as his symptoms caused clinically significant impairment, but the nature of the underlying condition was not clear. The differential diagnosis of this patient’s symptoms included primary psychiatric etiologies such as monopolar or bipolar depressionand schizophrenia-spectrum diagnoses, as well as catatonic syndrome due to another medical condition. The differential for medical causes of catatonia is broad, including endocrinological disruptions; hepatic or uremic encephalopathy; vitamin B_12_ deficiency; inflammatory disorders such as anti-NMDA receptor encephalitis, systemic lupus erythematosus, or multiple sclerosis; metabolic disorders such as Wilson disease or acute intermittent porphyria; neurological causes including head trauma, seizure disorders, and space-occupying lesions; and infectious causes such as human immunodeficiency virus (HIV) encephalopathy, HSV encephalitis, or Lyme neuroborreliosis. In essentially all of these conditions, acute-onset catatonic syndrome in the absence of significant medical findings would constitute an atypical presentation.

Despite extensive workup, the results failed to point toward any clear indication of the presence of organic disease. We did not pursue a repeat brain MRI with contrast due to the risk of gadolinium exposure, paired with improvement in the patient’s symptoms. As the patient’s clinical symptoms resolved with empirical treatment of catatonia, additional history supported the likelihood of a primary psychiatric process as the most likely etiology of the catatonic episode, with a potential contribution of risk by regular cannabis use (for example, [18, 19]).

### Treatment

Treatment with lorazepam and diazepam produced a partial and temporary resolution in symptoms. The patient was adherent to treatment as assessed by reports and documentation provided by nursing staff on the inpatient unit. No adverse events were noted by staff or reported by the patient. However, the patient’s symptoms continued to be life-threatening due to the inability to maintain adequate oral intake, and thus we chose to initiate the second-line treatment, electroconvulsive therapy (ECT). The patient underwent his first treatment with bilateral brief-pulse ECT on hospital day 24. Immediately upon recovery from anesthesia, the patient exhibited a sharp return of his faculties with greatly improved (though still limited) insight into his condition. He repeatedly asked why he was in the hospital and had a limited memory of the events of the last several weeks. The latency in response to questioning, rate of spontaneous speech, and question generation were drastically improved. Orientation, insight, and memory formation remained limited.

Over the next 1.5 days, the patient’s symptoms returned, with a near relapse to his prior catatonic state by the morning prior to his second ECT session on hospital day 26. Upon recovery, he again displayed increased awareness and confusion with respect to recent events. He was oriented to his name and birthdate, presence in a hospital, and approximate time of year (winter). He was able to roughly estimate his time in the hospital as “a few weeks.” Over the weekend following this treatment, the patient remained largely improved, though he reported feeling confused and displayed mild to moderate psychomotor retardation, with continued lack of insight into the reason for his hospitalization. The return of his symptoms was less pronounced after his second ECT treatment as compared with the first.

Upon recovery from his third ECT treatment on hospital day 29, the patient exhibited a return of insight, orientation, and personality. He reported mental “cloudiness” and short-term memory issues most notable within the 24 hours after each session, consistent with common side effects reported by many patients undergoing ECT, and these resolved after his final session. He denied headache, nausea, or other symptoms associated with ECT. The patient underwent a total of six sessions of ECT, with discontinuation of benzodiazepines for 12 hours prior to each session.

Additional history was obtained from the patient after his confusion and disorientation dissipated. He indicated that, in the months prior to his hospitalization, he had experienced depressed mood, low self-esteem, amotivation, anhedonia, weight loss, and reduced energy. In the days before his hospitalization, he remembered seeing dark shadows and hearing voices that were self-critical. He also reported regular usage of cannabis over the previous 3–4 years, varying between daily to once-weekly usage over this time. In the approximate month prior to his hospitalization, he did not use cannabis, consistent with his toxicology screen at presentation and roommate report. He reported only occasional social alcohol usage and no additional drug use.

When asked about previous psychiatric history, the patient endorsed symptoms that occurred the year prior to his current hospitalization (also in winter months) that were consistent with a major depressive episode,  though he had not been professionally evaluated for these symptoms. Interestingly, the patient described symptoms of psychomotor retardation during this prior episode that, while much milder in severity, were reminiscent of his current presentation. The patient denied any history of symptoms that would characterize mania or psychosis.

After completion of the ECT course, the patient was started on lithium for a presumed diagnosis of either bipolar disorder, depressive episode with catatonia; or major depression with catatonia. He was referred for outpatient psychotherapy and medication management on discharge to further assess and determine whether he may respond to standard antidepressant therapy.

The patient was discharged from the hospital on hospital day 40. On the day of discharge, he was able to converse readily with the treatment team, and he no longer displayed any observable symptoms of catatonia. He experienced memory loss for the immediate hours after each ECT treatment and remained mildly disoriented to his situation. However, he was able to interact with members of the care team in a casual fashion, interjecting humor into his daily interview. He was released to home with his family. The patient was seen 8 days after discharge by an outpatient psychiatrist. By that time he was able to describe a much clearer history of prior depressive episodes without manic episodes. He was given a diagnosis of major depressive disorder with catatonia and mood-congruent psychotic features. Also on the differential were bipolar disorder and schizoaffective disorder, though there was not sufficient evidence at this time for any history of manic episodes or psychotic symptoms outside of mood episodes.

## Conclusions

When presented with a case of idiopathic or unspecified catatonia, clinicians must maintain a high index of suspicion that the underlying disease process could be of nonpsychiatric origin. This is especially true in cases of young patients without a history of psychiatric illness, as higher frequencies of “organic” causes have been described in children and adolescents with catatonia [20]. The patient described in this report was transferred to our facility with a large medical workup already undertaken, in addition to treatment with benzodiazepines, to which he exhibited a partial response. To ensure that the differential diagnosis of the patient’s condition was thoroughly explored, we reconsidered and followed up with many of the test results that had been provided.

In particular, the diagnosis of Lyme neuroborreliosis warranted further consideration. This possibility was ruled out by providers at the first hospital after the CSF PCR results were found to be negative. However, serological testing has a much higher sensitivity for detecting Lyme neuroborreliosis as compared with PCR analysis of CSF [21], and false negative rates are high in CSF PCR analysis [22]. While catatonia is not a common manifestation of Lyme neuroborreliosis, previous case studies have been described [23]. In the context of sufficient clinical suspicion for Lyme disease, lack of significant response to benzodiazepine treatment, and the relative safety of the indicated treatment, we initiated doxycycline (300 mg/day) on hospital day 17 and ordered confirmatory tests. immunoglobulin (Ig)G Lyme serology results did not confirm a Lyme disease diagnosis, and doxycycline was discontinued on day 23 due to negative results and lack of significant clinical improvement after 6 days of treatment. Of note, the following bands were found on Western blot: hospital day 2, IgM: p41, p23; IgG: p30, p41, p66; hospital day 17: IgM: p41; IgG: p41, p66, p93. While these findings do not meet the Centers of Disease Control and Prevention (CDC) criteria for a diagnosis of Lyme disease, several of these bands are considered to be specific for *Borrelia burgdorferi* [24]. Furthermore, IgM to IgG seroconversion is highly variable and may take longer than the period between serial tests in our patient [25]. As such, the diagnosis of Lyme disease in this patient cannot be conclusively ruled out, and it is possible that a subclinical infection could have contributed to the onset of this patient’s presentation. Nonetheless, in the absence of fever, arthritides, and serological signs of systemic infection, and in combination with history obtained after symptomatic resolution, a psychiatric etiology remains most likely in this case.

Regardless of the mechanism of disease, catatonic symptoms often show resolution with benzodiazepines and/or ECT. Approximately 80% of catatonic patients experience relief of symptoms within minutes of benzodiazepine administration, and the remaining are nearly all relieved by ECT [26–31]. It has been proposed that ECT should be preferred to benzodiazepines as a first-line treatment, as the effect of the latter on catatonic symptoms is transient and does not relieve every sign/symptom of an acute catatonic stupor [32, 33]. The mechanism by which induced seizures produce a resolution of catatonia is unknown, though viable hypotheses have been proposed that implicate release of neuroendocrine hormones, gliosis, and neurogenesis [34, 35]. Additional research should be directed towards identifying the key mechanisms of therapeutic effect in the interest of optimizing treatment protocols for catatonia.

The presentation of an acute onset of psychomotor symptoms in the absence of a history of mental illness warrants extensive workup to rule out medical causes to identify the appropriate course of treatment. An inability to obtain an appropriate history from the patient may complicate this task further. In the case of catatonia, it is fortunate that a number of effective treatments are available to achieve immediate and potentially lasting resolution of symptoms. These gold standard treatments can be initiated during the course of ongoing diagnostic workup, and patients’ responses to treatment can serve as additional information to support a given diagnosis.

### Teaching points

- Catatonia is a complex psychiatric syndrome defined by 3 or more of 12 psychomotor features, typically predominated by slowness of movement, decreased engagement with surroundings, and peculiar motor activity.
- Catatonia is not considered an independent class in the DSM-5 but is recognized as (1) catatonia associated with another mental disorder (that is, neurodevelopmental, psychotic, bipolar, depressive, and other disorders), (2) catatonia due to another medical condition, and (3) unspecified catatonia.
- Before catatonia is used as a specifier for a mental disorder, a wide variety of other medical conditions need to be ruled out. These conditions include, but are not limited to, medical conditions due to infectious, metabolic, and neurologic conditions.
- In the absence of a treatable underlying medical condition, the treating physician should initiate empirical therapy. Treatment with benzodiazepines (80% effective) and ECT (80–100% effective) often produces at least temporary resolution of symptoms immediately and can allow for improved communication with the patient and time for clinical decision-making.

## Data Availability

The clinical data and test results described in this article are protected by patient confidentiality regulations. Limited de-identified results are available from the corresponding author on reasonable request.
